# For the Medical and Physical Journal

**Published:** 1814-06

**Authors:** 


*For the Medical and Physical Journal.*

IN the account of the late trial of Major Gordon for the murder of a private in the 2d regiment of guards, by holding in his hand a sword on which the deceased fell, Messrs. Snowden and Plake give their medical evidence, stating that from the appearance of the wound they will take upon themselves to declare, that it must have been inflicted by the body falling upon the sword, and not occasioned by a thrust of the weapon. Having paid some attention to the different appearances of wounds of this nature, I must own I have have not been able to discover, nor can I conceive, the difference occasioned by a sharp sword thrust into the body, or the body forced against the sword. If this should meet their eye, they will probably explain to the public the reasons which induced them to give this extraordinary evidence, which, if satisfactory, will contribute a valuable fact to the science of medical jurisprudence.

CHIRURGUS.

---

*For the Medical and Physical Journal.*

